# Regulation of global gene expression and cell proliferation by APP

**DOI:** 10.1038/srep22460

**Published:** 2016-03-03

**Authors:** Yili Wu, Si Zhang, Qin Xu, Haiyan Zou, Weihui Zhou, Fang Cai, Tingyu Li, Weihong Song

**Affiliations:** 1 Chongqing City Key Lab of Translational Medical Research in Cognitive Development and Learning and Memory Disorders, Children’s Hospital of Chongqing Medical University, Chongqing, 400014, China; 2 Ministry of Education Key Lab of Child Development and Disorders, Children’s Hospital of Chongqing Medical University, Chongqing, 400014, China; 3 Townsend Family Laboratories, Department of Psychiatry, Brain Research Center, The University of British Columbia, 2255 Wesbrook Mall, Vancouver, BC V6T 1Z3, Canada; 4 Department of Psychiatry, Jining Medical University, Jining, Shandong, 272067, China

## Abstract

Down syndrome (DS), caused by trisomy of chromosome 21, is one of the most common genetic disorders. Patients with DS display growth retardation and inevitably develop characteristic Alzheimer’s disease (AD) neuropathology, including neurofibrillary tangles and neuritic plaques. The expression of amyloid precursor protein (APP) is increased in both DS and AD patients. To reveal the function of APP and elucidate the pathogenic role of increased APP expression in DS and AD, we performed gene expression profiling using microarray method in human cells overexpressing APP. A set of genes are significantly altered, which are involved in cell cycle, cell proliferation and p53 signaling. We found that overexpression of APP inhibits cell proliferation. Furthermore, we confirmed that the downregulation of two validated genes, PSMA5 and PSMB7, inhibits cell proliferation, suggesting that the downregulation of PSMA5 and PSMB7 is involved in APP-induced cell proliferation impairment. Taken together, this study suggests that APP regulates global gene expression and increased APP expression inhibits cell proliferation. Our study provides a novel insight that APP overexpression may contribute to the growth impairment in DS patients and promote AD pathogenesis by inhibiting cell proliferation including neural stem cell proliferation and neurogenesis.

Down syndrome (DS), caused by trisomy of chromosome 21, is one of the most common genetic disorders. Patients with DS display growth retardation, cardiac and gastrointestinal abnormalities, immune system defects and inevitably develop characteristic Alzheimer’s disease (AD) neuropathology including neuritic plaques and neurofibrillary tangles. Neuritic plaques, the unique feature of AD neuropathology, mainly consist of amyloid-beta protein (Aβ)^1^,^2^. Aβ is derived from amyloid precursor protein (APP) after sequential cleavages by β- and γ-secretase. APP expression is increased in both DS patients and AD patients, contributing to an increase of Aβ generation and neuritic plaque formation^3^,^4^,^5^,^6^,^7^,^8^,^9^,^10^. However, in addition to promoting Aβ generation and neuritic plaque formation, the pathogenic role of increased APP expression in DS and AD remains elusive.

The *APP* gene, located on chromosome 21, spans approximately 290,586 bp of genomic DNA. It is ubiquitously expressed in a wide variety of human tissues and highly expressed in human brains. APP, a type I transmembrane protein, is predominantly cleaved by α-secretase, generating an N-terminal secreted soluble APPα (sAPPα) and a C-terminal 83-amino acid fragment (C83). C83 is further cleaved by γ-secretase to release a 3 kDa fragment (P3) and APP intracellular domain (AICD)^11^,^12^,^13^,^14^. The minority of APP is cleaved by β-site APP cleaving enzyme 1 (BACE1), the β- secretase *in vivo*, to generate a C-terminal fragment of 99 amino acids or 89 amino acids (C99 or C89), which will be further cleaved to generate Aβ or truncated Aβ and AICD by γ-secretase^15^,^16^,^17^,^18^,^19^. In addition, APP is also cleaved by BACE2 (beta-site APP cleaving enzyme 2), a θ-secretase, to generate a C-terminal fragment of 80 amino acids followed by γ-secretase cleavage^20^,^21^,^22^.

In addition to Aβ generation and neuritic plaque formation, additional functions of APP have been implicated. For example, APP knockout mice display various abnormalities, such as reduced body weight and size, increased frequency and severity of kainite-induced seizures, and deficits in learning and memory^23^,^24^,^25^,^26^. Moreover, triple knockout of APP and its two homologs, APLP1 and APLP2, is early postnatal lethal, indicating that APP, APLP1 and APLP2 are functionally redundant to certain extent and play a key role in survival^27^. sAPPα also shows neurotrophic property^27^,^28^,^29^,^30^,^31^. AICD is not only involved in APP trafficking but also plays a potential role in transcription regulation via its interaction with Fe65 and Tip60^32^,^33^,^34^. APP may serve as a cell surface receptor mediating the signal transduction and Aβ is one of the ligands^35^,^36^,^37^. Furthermore, APP/APP interaction plays an important role in cell and synaptic adhesion^38^,^39^. Interestingly, the role of APP in cell proliferation is controversial due to the different APP fragments and mouse strains used in the studies^40^,^41^,^42^,^43^,^44^,^45^,^46^,^47^,^48^. Recently, we reported that overexpression of APP promotes stress-induced apoptosis^10^. Taken together, the evidence indicates that APP is a multifunctional protein by itself and the proteolytic fragments of APP also possess major cellular functions.

APP expression is increased in both DS patients and AD patients^9^. However, few studies have addressed the pathogenic role of increased APP expression in DS and AD pathogenesis other than its role in promoting Aβ generation and neuritic plaque formation. Although gene expression profiles of transgenic mice carrying Swedish mutant APP have been reported^49^, the effect of increased wildtype APP expression in DS and some sporadic AD cases on gene regulation remains unknown. To investigate the gene expression profiles in human cells overexpressing wildtype APP, we performed the microarray experiment followed by qRT-PCR to validate the gene expression. We found that the expression of 2304 genes are significantly altered, which distribute on all chromosomes. In addition, 197 gene ontology (GO) categories of biological processes and 13 canonical pathways are significantly affected, including basal metabolism, cell cycle and cell proliferation etc. Moreover, we demonstrated that APP overexpression leads to proliferation impairment in human cells and the downregulation of two identified genes, PSMA5 and PSMB7 may play a role in APP overexpression –induced proliferation impairment.

## Results

### Transcriptional profiling in APP overexpression cells

**Figure 1:**
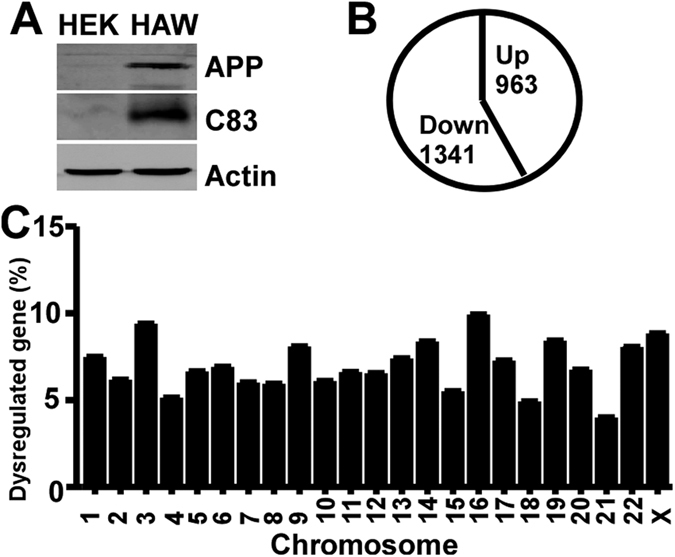
Transcriptional profiling in APP overexpression cells. (**A**) Cell lysates from HEK293 and HAW cells were resolved on 10% Tris-Glycine or 16% Tris-Tricine SDS-PAGE. Full-length APP and C-terminal fragment of APP with 83 amino acids (C83) were detected by C20 antibody. β-actin served as internal control was detected by mouse anti-actin antibody (Sigma). (**B**) Amplified RNA from HEK293 and HAW cells was used to perform whole genome expression assay with illumina system. At ±1.49-fold cut-off, 2304 genes were significantly regulated in HAW cells, p < 0.05, analyzed by Beadstudio software. (**C**) Chromosome distribution of significantly altered genes in HAW cells. Values represent the percentage of differentially expressed genes on each chromosome at ±1.49-fold cut-off.

To investigate the role of APP overexpression in the regulation of global gene expression, the wildtype human APP was stably transfected into HEK 293 cells as HAW cells. Compared with parental HEK293 cells, exogenous full-length APP and C-terminal fragment of APP with 83 amino acids (C83) were detected by C20 antibody which recognizes the last 20 amino acids of APP C-terminal (Fig. 1A). Whole genome expression assay was performed to profile the differential gene expression between HAW and HEK293 cells. Compared with HEK293 cells, 3003 genes of total 37847 illumina gene IDs were significantly altered in HAW cells at p < 0.05, including 1240 upregulated genes and 1763 downregulated genes. The normalization, background extraction and illumina custom false discovery rate correction were applied. The expression of 2491 genes is significantly changed at ±1.49 fold cut-off. 2304 genes including 963 upregulated genes and 1341 downregulated genes were counted when unidentified gene symbols and genes on unknown chromosome were ignored (Fig. 1B).

It was reported that the differentially expressed genes in DS patients distribute on all chromosomes, ranging from 3–6.6%, except for 27% genes on chromosome 21^50^. However, the effect of each individual gene on global gene expression is unknown. Although studies suggested that APP may play an important role in gene transcription regulation via sAPP, Aβ or AICD, no study has examined the transcriptional profiling of APP in human cells. Here we specifically investigated the role of APP in whole genome expression in human cells. The differentially expressed genes distributed on all 23 chromosomes, ranging from 20 to 234 genes on each chromosome. Since the number of genes on each chromosome has huge difference, we presented the percentage of differentially expressed genes on each chromosome. There are 7.49%, 6.18%, 9.41%, 5.15%, 6.65%, 6.91%, 6.01%, 5.94%, 8.09%, 6.11%, 6.62%, 6.55%, 7.41%, 8.38%, 5.52%, 9.93%, 7.28%, 4.93%, 8.43%, 6.75%, 4.00%, 8.07% and 8.48% significantly altered genes on chromosome 1to X, respectively (Fig. 1C). The data indicate that APP has global effect on gene transcription, covering all chromosomes.

### Gene ontology, canonical pathway and network analysis

**Figure 2:**
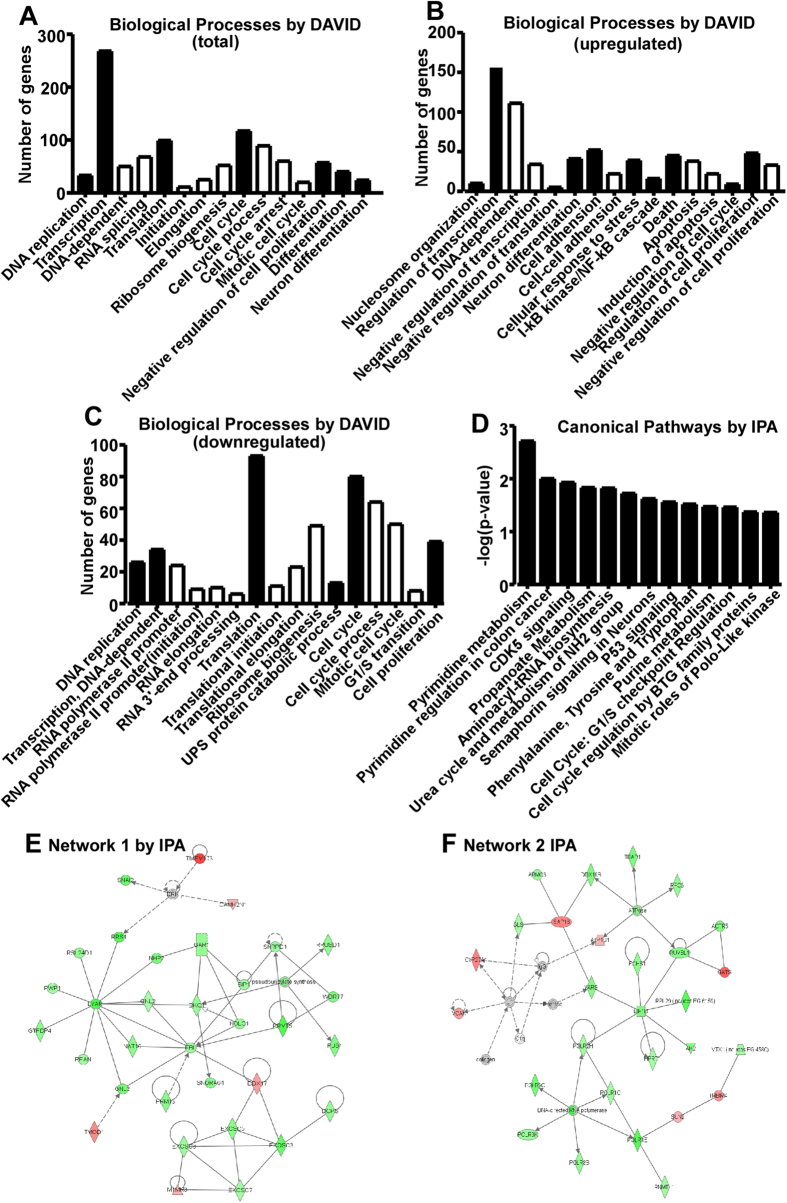
Gene ontology, canonical pathway and network analysis. (**A**) Significantly affected GO categories (black bar) and subcategories (white bar) of biological process are plotted, p < 0.05 by DAVID. (**B**) Significantly affected GO categories (black bar) and subcategories (white bar) by upregulated genes are plotted, p < 0.05 by DAVID. (**C**) Significantly affected GO categories (black bar) and subcategories (white bar) by downregulated genes are plotted, p < 0.05 by DAVID. (**D**) Canonical pathway analysis was performed by using IPA. 13 significant affected pathways are generated, p < 0.05 (-log p-value > 1.30). According to the functional and biological connectivity, 25 networks are constructed by IPA. Two of top ranked networks, (**E**) RNA post-transcriptional Modification/Cancer/Cell Cycle network and (**F**) nucleic acid metabolism/small molecule biochemistry/genetic disorder network, generated by IPA are presented. Red and green colored genes represent up- and downregulated genes, respectively. Solid lines and dash lines denote direct or indirect interactions, respectively.

To reveal the potential function of differentially expressed genes by APP overexpression in HAW cells, the enrichment analysis of gene ontology (GO) was performed by DAVID, a web-based software for gene expression analysis. 2169 genes were identified by DAVID, including 910 upregulated and 1259 downregualted genes. 197 GO categories of biological processes were significantly affected (p < 0.05), including DNA replication (33 genes), transcription (268 genes), translation (99 genes), cell cycle (117 genes), negative regulation of cell proliferation (57 genes), cell morphogenesis involved in differentiation (40 genes), and regulation of neuron differentiation (24 genes) etc. (Fig. 2A). Subcategories of transcription (DNA-dependent transcription and RNA splicing), translation (initiation, elongation and ribosome biogenesis), and cell cycle (cell cycle process, mitotic cell cycle and cell cycle arrest) were also significantly affected (Fig. 2A). To further dissect the function of dysregulated genes, GO enrichment analysis was performed on upregulated and downregulated genes, respectively. The upregualted genes were involved in neucleosome organization (10 genes), regulation of transcription (156 genes), negative regulation of translation (5 genes), neuron differentiation (41 genes), cell adhesion (52 genes), cellular response to stress (39 genes), positive regulation of I-kappaB kinase/NF-kappaB cascade (16 genes), death (45 genes), negative regulation of cell cycle (9 genes) and regulation of cell proliferation (48 genes) etc.141 biological processes, p < 0.05. The subcategories of some processes are also involved (Fig. 2B). On the other hand, the downregulated genes were involved in 222 biological processes (p < 0.05), particularly including DNA replication, DNA-dependent transcription, translation, ubiquitin-dependent protein catabolic process, cell cycle, cell proliferation and their subcategories (Fig. 2C). Moreover, total altered genes, upregulated genes and downregulated genes were involved in 70, 17 and 74 GO categories of molecular function, respectively (p < 0.05). 63, 37 and 73 GO categories of cell component were significantly affected by total, upregulated and downregulated genes,respectively (p < 0.05).

GO enrichment analysis only presents each function independently. Pathway analysis considers the function dependency and molecular interaction^51^. Thus we performed canonical pathway analysis by using IPA. There are 13 significant affected pathways (p < 0.05), including pyrimidine metabolism, polyamine regulation in colon cancer, CDK5 signaling, propanoate metabolism, aminoacyl-tRNA biosynthesis, urea cycle and metabolism of amino groups, semaphoring signaling in neurons, p53 signaling, phenylalanin/tyrosine/tryptophan biosynthesis, purine metabolis, cell cycle-G1/S checkpoint regulation, cell cycle regulation by BTG family proteins and mitotic roles of Polo-like kinase (Fig. 2D). Based on the functional and biological connectivity, 25 networks were generated by IPA. Two of the top ranked networks were presented, which are RNA post-transcriptional modification/cancer/cell cycle network with the score of 45 and 33 associated genes (Fig. 2E), and nucleic acid metabolism/small molecule biochemistry/genetic disorder network with the score of 33 and 28 associated genes (Fig. 2F). The data highly suggested that the basal metabolic rate, cell cycle and cell proliferation are dysregulated by APP overexpression.

### Alteration of the genes involved in basal metabolism, cell cycle and cell proliferation

**Figure 3:**
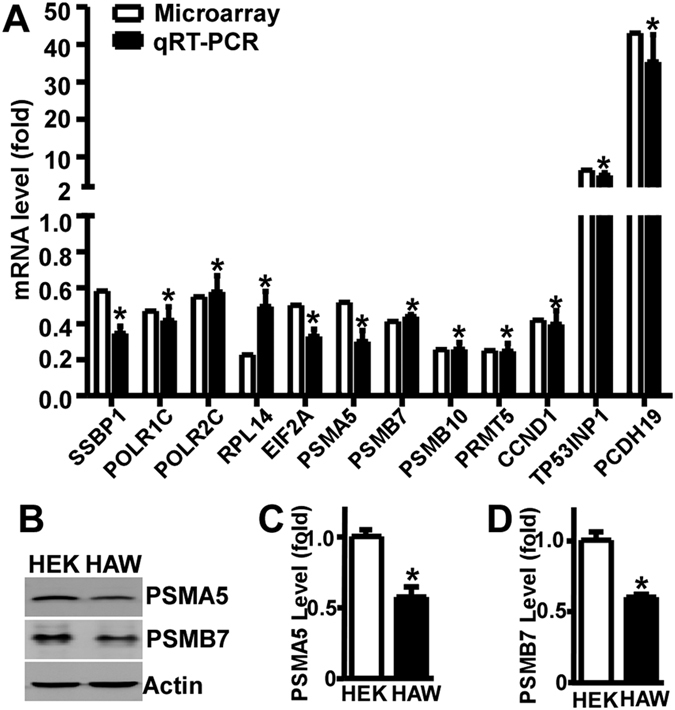
Validation of gene expression. (**A**) Significantly altered genes identified by microarray experiments (white bar) were validated by qRT-PCR (black bar) at mRNA level. (**B**) The expression of two identified genes, PSMA5 and PSMB7, were validated at protein level. Lysates of HEK293 and HAW cells were subjected to Western blot analysis. PSMA5 or PSMB7 was detected by PSMA5 or PSMB7 antibody. β-actin served as internal control was detected by mouse anti-actin antibody. (**C**,**D)** Quantification of (**B**). Values represent mean ± SEM. N > = 3, *p < 0.05 by student *t*-test.

To confirm the altered gene expression by APP in microarray experiments, quantitative PCR following reverse transcription (qRT-PCR) was performed by using three sets of independent samples. As GO enrichment analysis and pathway analysis clearly showed that APP overexpression significantly affected DNA replication, transcription, translation, cell adhesion, cell cycle and cell proliferation, 12 genes involved in these GO categories and pathways were selected to be validated. Data from microarray experiments showed that the expression of SSBP1 (DNA replication), POLR1C (transcription), POLR2C (transcription), RPL4 (translation), EIF2A (translation), PSMA5 (cell cycle), PSMB7 (cell cycle), PSMB10 (cell cycle), PRMT5 (cell proliferation) and CCND1 (cell cycle) was reduced to 0.58, 0.47, 0.55, 0.23, 0.50, 0.52, 0.41, 0.26, 0.25 and 0.42 fold, respectively, while the expression of TP53INP1 (cell cycle arrest and apoptosis) and PCDH19 (cell adhesion) was increased to 6.48 and 53.06 fold. Consistently, qRT-PCR results showed that the expression of SSBP1, POLR1C, POLR2C, RPL4, EIF2A, PSMA5, PSMB7, PSMB10, PRMT5 and CCND1 was reduced to 0.35 ± 0.04, 0.42 ± 0.08, 0.58 ± 0.09, 0.50 ± 0.09, 0.33 ± 0.04, 0.30 ± 0.06, 0.44 ± 0.01, 0.26 ± 0.04, 0.25 ± 0.04 and 0.40 ± 0.08 fold, respectively, while the expression of TP53INP1 and PCDH19 was increased to 5.11 ± 0.69 and 35.35 ± 7.24 fold (P < 0.05) (Fig. 3A). To further confirm the gene expression at protein level, the expression of two identified genes, PSMA5 and PSMB7, was examined by Western blot (Fig. 3A). The levels of PSMA5 and PSMB7 in HAW cells were markedly reduced to 0.57 ± 0.03 and 0.60 ± 0.02 fold, respectively (p < 0.05) (Fig. 3C,D). The alteration of the genes involved in basal metabolism, cell cycle and cell proliferation suggested that APP overexpression may affect cell proliferation.

### APP overexpression inhibits cell proliferation and PSMA5 and PSMB7 downregulation is involved in proliferation impairment

**Figure 4:**
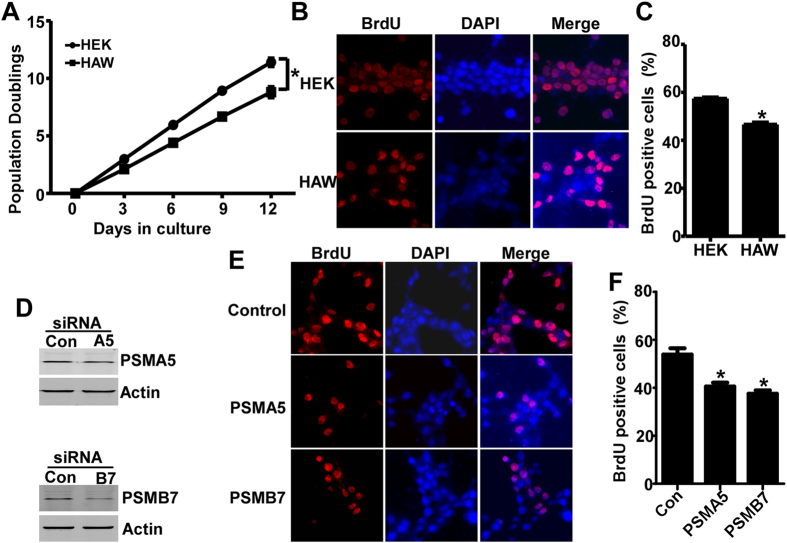
APP overexpression inhibits cell proliferation and the downregulation of two identified genes is involved in proliferation impairment. (**A**) Growth curve of HEK293 cells and HAW cells. Equal number of cells was seeded on 35 mm dishes and the cell number was counted every 3 days. The growth curve was plotted after 12 day’s culture. Values represent mean ± SEM. N = 3, *p < 0.05 by ANOVA. (**B**) BrdU cell proliferation assay of HEK293 and HAW cells. After 6-hour incubation with BrdU, BrdU incorporation was detected by BrdU antibody followed by cy3-conjugated anti-goat IgG (Red). Nuclei were stained with DAPI (Blue). (**C**) Quantification of BrdU positive cells in HEK293 and HAW cells. The percentage of BrdU-positive cells in each line was presented. Values represent mean ± SEM. N > = 3, *p < 0.05 by student *t*-test. (**D**) 3 days after scramble, PSMA5 or PSMB7siRNA transfection, the expression of PSMA5 or PSMB7 was examined by Wstern blot. PSMA5 or PSMB7 was detected by PSMA5 or PSMB7 antibody. β-actin served as internal control was detected by mouse anti-actin antibody. (**E**) 3 days after scramble, PSMA5 or PSMB7siRNA transfection, BrdU cell proliferation assay was performed. BrdU incorporation was detected by BrdU antibody followed by cy3-conjugated anti-goat IgG (Red). Nuclei were stained with DAPI (Blue). (**F**) Quantification of BrdU positive cells. The percentage of BrdU-positive cells in each line was presented. Values represent mean ± SEM. N > = 3, *p < 0.05 by student *t*-test.

To assess the effect of APP on cell growth, the number of cell population doublings (PDs) was examined in both HEK293 and HAW cells, and the growth curve was plotted after 12 day’s culture. The growth rate of the HAW cells was 77.18% of HEK293 cells, 8.80 ± 0.46 PDs vs. 11.40 ± 0.36 PDs (p < 0.05) (Fig. 4A). To further examine the effect of APP on cell cycle and cell proliferation, the rate of BrdU uptake in HAW cells and HEK293 cells was measured (Fig. 4B). After 6 hours incubation, cells labeled with BrdU were significantly less in HAW cells than those in HEK293 cells, 46.61 ± 0.87% vs. 57.46 ± 0.53% (p < 0.05) (Fig. 4C).

To determine whether APP-induced inhibition of cell proliferation is mediated by the dysregulated genes identified by microarray and qRT-PCR, knockdown experiments were performed in HEK293 cells by using siRNAs of two identified genes involved in cell cycle and cell proliferation, PSMA5 and PSMB7, respectively. Three days after PSMA5 or PSMB7 siRNA transfection, the expression of PSMA5 or PSMB7 was markedly reduced compared with that in scrambled siRNA transfected cells (Fig. 4D). The BrdU cell proliferation assay was performed following PSMA5 and PMB7 knockdown (Fig. E). After 6 hours incubation with BrdU, less cells were labeled with BrdU in PSMA5 or PMSB7 knockdown cells compared with cells transfected with scrambled siRNA, 40.54 ± 1.63% or 37.54 ± 1.43% vs. 53.87 ± 2.64%, (p < 0.05) (Fig. 4F). The data indicate that the downregualtion of PSMA5 and PSMB7 is involved in APP overexpression-induced inhibition of cell proliferation.

## Discussion

Dysregulation of gene expression has been implicated in DS^10^,^52^,^53^,^54^,^55^,^56^. APP expression is increased in both DS patients and AD patients and increased APP expression could play multiple roles in the pathogenesis of DS and AD in addition to the increase in Aβ generation and neuritic plaque formation. A recent study showed that wildtype human APP overexpression promotes cognitive deficits in mice which is unrelated to Aβ^57^. To profile the whole genome expression by APP overexpression could reveal the function of APP and elucidate the pathogenic role of increased APP expression. Previously, gene expression profiles in APPswe transgenic mice, DS mouse model and DS human brains have been reported^49^,^50^,^58^,^59^. However, it should be considered that data from mouse cannot fully represent changes in human. Moreover, APP with Swedish mutation is processed differently compared with wildtype APP, and APPswe leads to early onset AD which is not evidenced in wildtype APP^15^. Thus, profiling the gene expression in human cells overexpressing wildtype APP would provide valuable information of the function of APP and the pathogenic role of APP overexpression in both DS and AD.

In this study we performed whole genome gene expression assay in a human cell line. 3003 genes of 37847 illumina genes are significantly altered, around 8% of total genes, indicating that APP has global effects on gene regulation, not limiting to a small number of genes. Moreover, the affected genes distribute on23 chromosomes, ranging from 4.00% to 9.93%. It suggests that increased APP expression relatively equally affects the gene regulation on all chromosomes with no specific chromosomal preference. In addition, APP overexpression does regulate the expression of genes on chromosome 21 (9 upregulated and 11 downregulated genes). APP overexpression can further increase the expression of 9 genes on chromosome 21, indicating that the expression level of genes on chromosome 21 could increase to more than 1.5 fold in DS. Meanwhile, APP also reduces the expression of another 11 genes on chromosome 21, which may partially explain that the expression level of a group of genes on chromosome 21 does not change in DS patients. The data is consistent a previous report that only 27% genes on chromosome 21 are differentially expressed in DS patients^50^.

GO enrichment analysis showed that the processes involved in basal metabolism, cell cycle, cell proliferation, and cell differentiation are affected by APP overexpression. Pathway analysis by IPA further supports the results of GO enrichment analysis, e.g. P53 signaling pathway which is involved in both cell cycle and cell proliferation. To further dissect the function of dysregulated genes, GO enrichment analysis was performed on upregulated and downregulated genes separately. We found that the upregulated genes are significantly involved in cell death, apoptosis and cell adhesion, which not only supports previous observations that APP overexpression promotes apoptosis and cell adhesion but also provides potential molecular mechanisms underlying these effects. Moreover, the upregulated genes are involved in negative regulation of transcription, translation, cell cycle and cell proliferation, while the downregulated genes are involved in transcription, translation, cell cycle process and cell proliferation. It highly suggests that APP overexpression may reduce cell basal metabolism and inhibit cell proliferation via upregulating or downregulating genes involved in aforementioned processes. Moreover, we demonstrated that the downregulation of PMSA5 and PMSB7 involved in cell cycle process, which may contribute to APP overexpression-induced proliferation impairment. Our data indicate that increased APP may contribute to the growth retardation and developmental delay in DS by inhibiting basal metabolism and cell proliferation.

## Methods

### Cell culture, transfection and drug treatment

Human embryonic kidney (HEK) 293 cells and stable cells were grown in Dulbecco’s modified Eagle’s medium supplemented with 10% fetal bovine serum, 1% sodium pyruvate, 1% L-glutamine and 1% penicillin/streptomycin (Invitrogen). HAW cells are HEK293 cells stably overexpressing wildtype APP. Transfections were performed with Lipofectamine 2000 (Invitrogen). Scramble siRNA and pre-designed siRNA of PSMA5 or PSMB7 were purchased from Applied Biosystems.

### Whole-genome gene expression assay and Real-time PCR

RNA was isolated from cells using TRI-Reagent (Sigma-Aldrich). Thermoscript Reverse Transcription kit (Invitrogen) was used to synthesize the first strand cDNA following the manufacturer’s instruction. cRNA was amplified and purified by using IlluminaTotalPrep RNA amplification kit (Life Technologies). 1.5 μg cRNA of each sample was used for whole-genome gene expression direct hybridization assay with humanHT-12 v4 Expression Beadchip (Illumina) following the manufacturer’s instructions. Real-time PCR was performed following the Taqman One-Step RT-PCR protocol using premade primers and probes for each gene (Applied Biosystems). Amplification and detection were performed with the Smart cycler II (Cepheid). Three independent samples were assayed for each group.

### Immunoblotting

Cells were lysed in RIPA-Doc lysis buffer (1% Triton X100, 1% sodium deoxycholate, 0.1% SDS, 0.15 M NaCl, 0.05 M Tris-HCl, pH 7.2) supplemented with protease inhibitors (Boehringer Mannheim). 50 μg or 100 μg of total protein were resolved on 10–12% Tris-Glycine SDS-PAGE or 16% tris-tricine SDS-PAGE and transfered to polyvindylidinefluoride (PVDF-FL) membranes followed by immunoblotting. Rabbit anti-APP antibody C20 was used to detect APP and its C-terminal fragments (CTFs)^60^. β-actin served as internal control was detected by mouse anti-actin antibody (Sigma). PSMA5 and PSMB7were detected by a rabbit anti-PSMA5 antibody (Cell signaling) and anti-PSMB7 antibody (Abgent). IRDye 800CW-labelled goat anti-mouse antibody and IRDye 680RD-labelled goat anti-rabbit antibody were used as secondary antibodies, which were visualized on the Odyssey system (LI-COR Biosciences).

### Cell growth and Bromodeoxyuridine (5-bromo-2’-deoxyuridine, BrdU) proliferation assay

To monitor the growth rate of HAW cells, 2.5 × 10^5^ cells were seeded on 35 mm dishes and the cell number was counted every three days. To monitor the proliferation ability, the cells were incubated with 10uM BrdU for 6 hours. After incubation, the cells were washed with PBS and fixed with 4% paraformaldehyde for 20 minutes at room temperature. 2 M HCl was applied for 30 minutes at 37 °C followed by neutralization for 30 minutes with 0.1 M sodium borate. After permeabilization with 0.2% Triton solution for 15 minutes at room temperature, cells were blocked with goat serum followed by 2-hour incubation with goat anti-BrdU antibody (Sigma). Cells were washed with PBS and incubated with Cy3-conjugated anti-goat IgG (Thermo Scientific) for 1 hour. After washed with PBS, cells were stained with 4, 6-diamidino-2-phenylindole (DAPI) (Sigma). Cell images were taken by fluorescent microscope (Axiovert200, Carl Zeiss Inc.).

### Statistical analysis

Student’s *t* test was performed for the quantification of immunoblotting and BrdU labeling. Values of *P  * < 0.05 were considered significant. Differential gene expression, biological processes, canonical pathways and networks were analysed by Beadstudio, DAVID and Ingenuity Pathway Analysis software.

## Additional Information

**How to cite this article**: Wu, Y. *et al.* Regulation of global gene expression and cell proliferation by APP. *Sci. Rep.* **6**, 22460; doi: 10.1038/srep22460 (2016).
